# Supplementary material for: Identification of genomic features that uniquely impact estrogen receptor alpha binding and its effects on gene expression in endometrial cancer
Source: Genome Res. 2026 Sep;36(9):1771–84. doi: 10.1101/gr.281792.125 (PMC13534285; doi:10.1101/gr.281792.125)
Supplement: Supplementary file 1 [file Supplemental_Figures.pdf]

Supplementary Information

Identification of genomic features that uniquely impact estrogen receptor alpha binding and its effects on gene expression in endometrial cancer

Alexandra Richey, Noel Kitchen, Craig M. Rush, Jeffery M. Vahrenkamp, Hosiana Abewe, Kyle S. Osbourne, Alex J. Bott, Brian K. Dalley, Jared Rutter, and Jason Gertz

Supplementary Methods

Estrogen response element identification and classification

To identify all EREs in the human female genome, the program Patser-v3e (Hertz and Stormo 1999) was used to scan the hg19 genome to find and score sequences that matched closely to the canonical ERE motif, as defined by a position-specific scoring matrix (PSSM) created from the binding sites of ER in multiple estrogen responsive cell lines (Gertz et al. 2013). The hg19 build of the human genome was used to align with previously published datasets in our analyses. We do not believe that realigning our data to the GRCh38 genome build would affect the results of this work, as we focused our analyses on uniquely alignable regions of the genome. Patser was run with the following flags "-c -u2 -v". Regions above the numerically calculated cutoff were kept for downstream analysis as EREs, and the score was kept as a representation of the log of the relative affinity that region has for binding ER. The default cutoff score is the score at which the probability of observing a score greater than or equal to the cutoff is equal to the adjusted information content (Guhathakurta 2006). The adjusted information content is calculated based on the divergence of frequency of each nucleotide within a sequence from the a priori frequency of each nucleotide (each nucleotide occurring 25% of the time) and adjusted based on the length of the consensus sequence (the ERE) and the size of the sequences being scanned (the human genome) (Hertz and Stormo 1999). Since both cell lines are female, any EREs found on the Y Chromosome were removed from this list. The list of EREs was intersected, using BEDtools intersect (Quinlan and Hall 2010), with published ER ChIP-seq data taken from either T-47D cells or Ishikawa cells (Gertz et al. 2013). Any ERE that intersected with an ER ChIP-seq peak was labeled as “bound”; otherwise, it was labeled as “unbound”. Then, significant genes from published bulk RNA-seq data (Gertz et al. 2013) and single-cell RNA-seq data (Ginley-Hidinger et al. 2024) were combined to create a list of estrogen responsive genes for each cell line. For the bulk RNA-seq with an 8-hour

estrogen treatment, any gene with an adjusted *P*-value of 0.05 or lower was included as an estrogen responsive gene. For the single-cell RNA-seq data, any gene that had an adjusted *P*-value of 0.05 or lower at any time point (2-, 4-, or 8-hour) of estrogen treatment compared to the 0-hour timepoint was included as an estrogen responsive gene. The final list of estrogen responsive genes was the union of genes from the bulk RNA-seq and the single-cell RNA-seq. A bound ERE within 100kb of the TSS of an estrogen responsive gene was labeled as "regulatory". A bound ERE within 5kb of the TSS of an estrogen responsive gene also received the designation of promoter-proximal, with all other regulatory EREs receiving the designation of promoter-distal.

Comparison of bound EREs in cell line models to bound EREs in patient-derived models

All significant EREs were intersected with ER ChIP-seq data from multiple breast and endometrial cancer patient samples (Rodriguez et al. 2020) using BEDtools intersect (Quinlan and Hall 2010). Then, bound EREs from each sample were intersected with the bound EREs in Ishikawa and T-47D cells. A Wilcoxon sign-ranked test was performed to compare the overlaps between each patient sample and cell line to determine if Ishikawa bound EREs and T-47D bound EREs were more similar to endometrial and breast cancer patient samples, respectively.

Creation of experimental-focused dataset and motif-focused dataset for feature selection analysis

For the initial data frames used, the relative affinity score given by Patser, ChIP-seq of multiple transcription factors and histone modifications, ATAC-seq, DNase-seq, and PRO-seq were compiled. For each ERE, the normalized reads per million of each ChIP-seq, ATAC-seq, DNase-seq, and PRO-seq dataset at the location of the ERE were calculated, and then each dataset was converted to *Z*-scores. See Supplemental Table 1 for information about all datasets. All data were from T-47D and Ishikawa cells grown in the absence of estrogen. HiChIP data was used to determine which bound EREs had a 3D genomic interaction with the 5kb window around the TSS of an estrogen responsive gene, and this data factor was labeled "Target Gene 3D Loop." HiChIP was also used to determine the number of 3D genomic interactions in which each ERE (bound or unbound) was involved. To prevent redundancy with the Target Gene 3D Loop data, we only counted interactions that did not involve the TSS of an estrogen responsive gene. This factor was called "Non-Target Gene Loops."

For the motif analysis, the comprehensive hg19 BED file from Homer that lists the locations of the cognate DNA binding motifs for 450 DNA-binding proteins was used (Heinz et al. 2010). BEDtools intersect (Quinlan and Hall 2010) was used to intersect this file with a BED file of the EREs in the human female genome with a 100bp window centered on each ERE. A binary classifier was then created for each motif. An ERE either had the motif in its 100bp window or it did not. Spearman correlation between each motif was calculated using the R command cor from the stats package (R Core Team 2016). Using the correlation values, clustering using hclust from the R stats package, with the average method, was performed, and the cluster tree was cut off at 0.50, reducing the list of motifs down to 270 motif clusters (Table S3). The binary classifier was updated to reflect whether an ERE 100 bp window had any of the motifs within each cluster. Motif clusters that contained a motif that matched at least an ERE half-site were not displayed or considered for further analysis.

Feature selection analysis using Boruta

The default settings were used for all Boruta runs, using the function Boruta from the R package Boruta (Kursa and Rudnicki 2010), except that the number of threads used was 20. For all initial runs, the entire dataset was used, meaning all EREs. In addition, Boruta was performed using down-sampling for predicting ER binding. All bound EREs were used with an equal number of randomly selected unbound EREs to account for the much larger number of unbound EREs. This was repeated 100 times, and the average importance scores and the percentage of runs where the factor was confirmed important by Boruta were calculated. Down-sampling was not used when running Boruta to evaluate predicting effects on expression, because the number of regulatory and nonregulatory EREs was similar.

Creation of receiver operating characteristic curves and logistic regression models

The command roc, from the package pROC (Robin et al. 2011), was used with default parameters to create the receiver operating characteristic curves (ROC) and calculate the area under the curve (AUC). The R package caret (Kuhn et al. 2020) was used to perform logistic regression. To set up training and testing groups, the command createDataPartition was used, with the training groups set as 75% of the data and testing groups as 25%, repeated for 100 iterations. To set parameters for the training, the command trainControl was used, using the index from

createDataPartition with the following parameters: the method was set to cross validation ("cv"), class probabilities were set to be calculated instead of just the final class (classProbs=T), the summary function was set to "twoClassSummary", meaning the ROC AUC, the sensitivity, and the specificity were designated as the metrics for assessing the performance of the model, and down-sampling was designated to account for the disparities in the dataset so that the training model used equal amounts of each type of ERE. The command train was used with the above training parameters, with logistic regression implemented using the options: method = "glm" and family = "binomial". The models returned the average and standard deviation of ROC AUC, sensitivity, and specificity from multiple iterations. This approach was used for creating models for both bound vs unbound EREs and regulatory vs. nonregulatory EREs, for all cell lines, with different variables depending on the application. Bound EREs were set to 1, and unbound EREs were set to 0; similarly, regulatory EREs were set to 1, and nonregulatory EREs were set to 0, with the logistic regression models using the default cutoff value of 0.5.

Generation of 3×ERE-driven GFP lentiviral plasmid

The pLU-CMV-GFP-pBLAST plasmid, a gift from Meenhard Herlyn (Kerafast, EW1034) (Roesch et al. 2010), was used as the destination lentiviral plasmid. pLU-CMV-GFP-pBLAST's CMV promoter was removed by restriction digest using ClaI (NEB, R0197S) and XbaI (NEB, R0145S), with 3'-phosphate groups removed using shrimp alkaline phosphatase (NEB, M0371S). The backbone was size selected using gel extraction (Qiagen, 28704). A 3×ERE sequence was PCR amplified from a donor plasmid, 3×ERE TATA Luc, a gift from Donald McDonnell (Addgene, 11354) (Hall and McDonnell 1999), using primers that added directional ClaI and XbaI restriction sites (Table S6). PCR products were digested using ClaI and XbaI, purified using DNA Clean and Concentrator (Zymo Research, D4014), and ligated into the pLU-CMV-GFP_pBLAST backbone. Ligation products were transformed into STBL3 chemically competent cells (Thermo Scientific, C737303) and selected on ampicillin (100 μg/mL) LB agar plates. Correct insertion of the 3×ERE sequence was determined using Sanger sequencing (Genewiz), and the resultant plasmid was termed pLU-3×ERE-GFP-pBLAST. Lentiviral particles were generated by co-transfection of pLU-3×ERE-GFP-pBLAST with helper plasmids pCMV-VSV-G, a gift from

Bob Weinberg (Addgene, 8454) (Stewart et al. 2003), and psPAX2, a gift from Didier Trono (Addgene, 12260) into HEK293T cells.

Ishikawa GFP reporter cell line creation

Ishikawa cells were transduced with 5 µg/mL polybrene (Santa Cruz Biotechnology, sc-134220) and the lentiviral particles containing the 3×ERE-driven GFP reporter at MOIs of 10-200. Cells were treated with 30 µg/mL blasticidin (Thermo Fisher Scientific, A1113903) initially to select for successfully transduced cells and then pooled. After 96 hours, the blasticidin dose was reduced to 10 µg/mL for maintenance, and 10 nM E2 was added to activate GFP. Cells were incubated in full media for 72 hours and then sorted for positive GFP expression using flow cytometry. GFP positive cells were plated at 1000 cells per 10cm plate to allow single cells to form distinct colonies for selection. Colonies were grown up to ~256 cells on media without blasticidin or E2, then selected and moved to individual wells of a 96 well plate. 10 µg/mL blasticidin was added to full media 48 hours after colonies were plated in 96 well plates and included in media for all further culturing of the cells.

To test GFP responsiveness, colonies were moved to hormone depleted media for 72 hours, then treated with 10 nM E2 or DMSO (vehicle) for an additional 72 hours. Then the GFP levels of the cells were measured using flow cytometry, and colonies with the largest difference in GFP levels between E2 and DMSO conditions were chosen to use for further experimentation. To increase the GFP fold change for the chosen colony, cells were treated with 100 µg/mL blasticidin for two weeks, and then retested for GFP responsiveness before being used in the CRISPR screen.

CRISPR epigenetic KO screen

The Human Epigenetic Knockout plasmid library (HEKL), which was a gift from Kivanc Birsoy (Addgene 162256) (Williams et al. 2020), was co-transfected with helper plasmids pCMV-VSV-G, a gift from Bob Weinberg (Addgene, 8454) (Stewart et al. 2003), and psPAX2, a gift from Didier Trono (Addgene, 12260), into HEK293T cells to generate lentiviral particles. Ishikawa cells with the 3×ERE GFP reporter were grown to confluency in 3 15cm plates. One plate was used to count cells, and the other two plates were transduced at an effective MOI of

0.3 with the HEKL lentiviral particles and 5 μg/mL polybrene in full media. Puromycin (Thermo Scientific, A1113803) was used at 1μg/mL in full media to select for successfully transduced cells 24 hours after transduction. Post puromycin selection, the cells were grown up to 10 million cells in full media with 10 μg/mL blasticidin, 2.5 million cells were frozen down for genomic DNA extraction and sequencing, and 2.5 million cells were moved to hormone depleted media with 10 μg/mL blasticidin for 96 hours. After 96 hours in hormone depleted media, 2.5 million cells were frozen for genomic DNA extraction and sequencing, and the remaining cells were treated with 10 nM E2 for 72 hours. Cells were then sorted using flow cytometry into four bins: negative GFP, low GFP, medium GFP, and high GFP. Genomic DNA was extracted from the cells in all four bins along with the cells from the post transduction pellet, and the post-hormone depleted media pellet using the Quick-DNA Miniprep Kit (Zymo, D3025). Libraries were constructed for sequencing the CRISPR sgRNA region using a two-step PCR process. Genomic DNA (100 to 5000 ng) was PCR amplified for 14 cycles using custom CRISPR PCR primers with NEBNext Ultra II Q5 Master Mix (NEB, M0544) (Table S6). This was performed as 8 replicate reactions with the amplicons being pooled before the next step. Dual unique indexing adapters (Illumina, 20027213) (Table S6) were added to the CRISPR amplicons during a second PCR with 6 cycles and performed as 3 replicate reactions. Libraries were pooled and sequenced on an Illumina NovaSeq X instrument as 150×150 cycle paired-end sequence runs. Two replicates, starting with transduction of the HEKL lentiviral particles, were performed.

Post-screen enrichment analysis using MAGeCK

The forward read FASTQ files, along with a guide RNA file, were analyzed using the MAGeCK program (Li et al. 2014; Williams et al. 2020). MAGeCK count was used to compile counts of each gRNA in each sample. This command allowed for combining two replicates to generate a single count file. MAGeCK test was used to test for enrichment. The first run designated the negative GFP bins as the control with the other three as the samples and the second run designated the negative, low, and medium GFP bins as the controls with the high GFP bin as the sample. After testing, the gene outputs were filtered by the number of gRNAs used in analysis, designated by MAGeCK as "good gRNAs." Genes included for future analysis had to have more than half of their gRNAs

included in the test analysis performed by MAGeCK. After filtering, the command p.adjust from the R stats package was used to adjust the *P*-values, using the "fdr" method. The negative significant genes from the negative GFP control analysis were labeled as ER enabling genes, and the positive significant genes from the negative, low, and medium GFP control analysis were labeled as ER blocking genes.

Ishikawa Cas9 cell line creation

The lentiCas9-Blast plasmid, a gift from Feng Zhang (Addgene, 52962) (Sanjana et al. 2014) was co-transfected with helper plasmids pCMV-VSV-G, a gift from Bob Weinberg (Addgene, 8454) (Stewart et al. 2003), and psPAX2, a gift from Didier Trono (Addgene, 12260), into HEK293T cells to generate lentiviral particles. Ishikawa cells were then transduced with 5 µg/mL polybrene and the lentiviral particles containing the 3×ERE-driven GFP reporter at MOIs of 0.5-10. Cells were treated with 30 µg/mL blasticidin initially to select for successfully transduced cells; after selection, the blasticidin dose was reduced to 10 µg/mL for establishment prior to cryo-preservation. Cas9 presence was verified using Western blot and an MOI of 5 was chosen.

Creation of ER and SOX17 targeting lentiviral plasmids

Guide RNAs (gRNAs) were designed to target *ESR1* and *SOX17* and ordered as single-stranded DNA primers from IDT (Table S6). For a control, we used the control sequence used previously (Sanjana et al. 2014). The LentiGuide-Puro plasmid, a gift from Feng Zhang (Addgene, 52963) (Sanjana et al. 2014), was used as the destination lentiviral plasmid. The plasmid was restriction digested using FastDigest Esp31 (Thermo Fisher Scientific, FD0454) with 3'-phosphate groups removed using shrimp alkaline phosphatase (NEB, M0371S). The backbone was size selected using 0.8% gel extraction. The gRNA primers were annealed in 10× T4 Ligation Buffer (NEB, B0202S) and phosphorylated using T4 Polynucleotide Kinase (NEB, M0201S) before being ligated to the destination plasmid using the Quick Ligation Kit (NEB, M2200S). Ligation products were transformed into STBL3 chemically competent cells (Thermo Scientific, C737303) and selected on ampicillin (100 µg/mL) LB agar plates. Correct insertion of gRNAs was determined using full plasmid sequencing (Plasmidsaurus). Lentiviral particles were generated by co-transfection of the LentiGuide Puro plasmids (with the gRNAs inserted) with

helper plasmids pCMV-VSV-G, a gift from Bob Weinberg (Addgene, 8454) (Stewart et al. 2003), and psPAX2, a gift from Didier Trono (Addgene, 12260) into HEK293T cells.

Western blotting

Western blotting was performed on Ishikawa control cells, Ishikawa Cas9 cells, *ESR1* KO cells, and *SOX17* KO cells as described previously (Rush et al. 2022) using the following primary antibodies: mouse anti-β-Actin (Santa Cruz Biotechnology, sc-47778), rabbit anti-Cas9 (Diagenode. C15310258), rabbit anti-ERα (Thermo Fisher Scientific, MA5-14501), goat anti-SOX17 (R&D Systems, AF1924). The following secondary antibody conjugates were used for detection: IRDye 680RD donkey anti-Mouse (Li-COR, 926-68072), IRDye 800CW donkey anti-rabbit (Li-COR, 926-32213), and IRDye 800CW donkey anti-Goat (Li-COR, 926-32214). Transfer onto nitrocellulose membranes (Thermo Scientific, IB33002X3) was performed using the iBlot 3 dry blotting system (Thermo Scientific, IB31001). Fluorescence signal from the antibodies were then detected using the Li-COR Odyssey Clx imaging system with the 800CW and 680RD channels. Western blot images were analyzed using Image Studio software (Li-COR).

Supplementary Figures and Tables

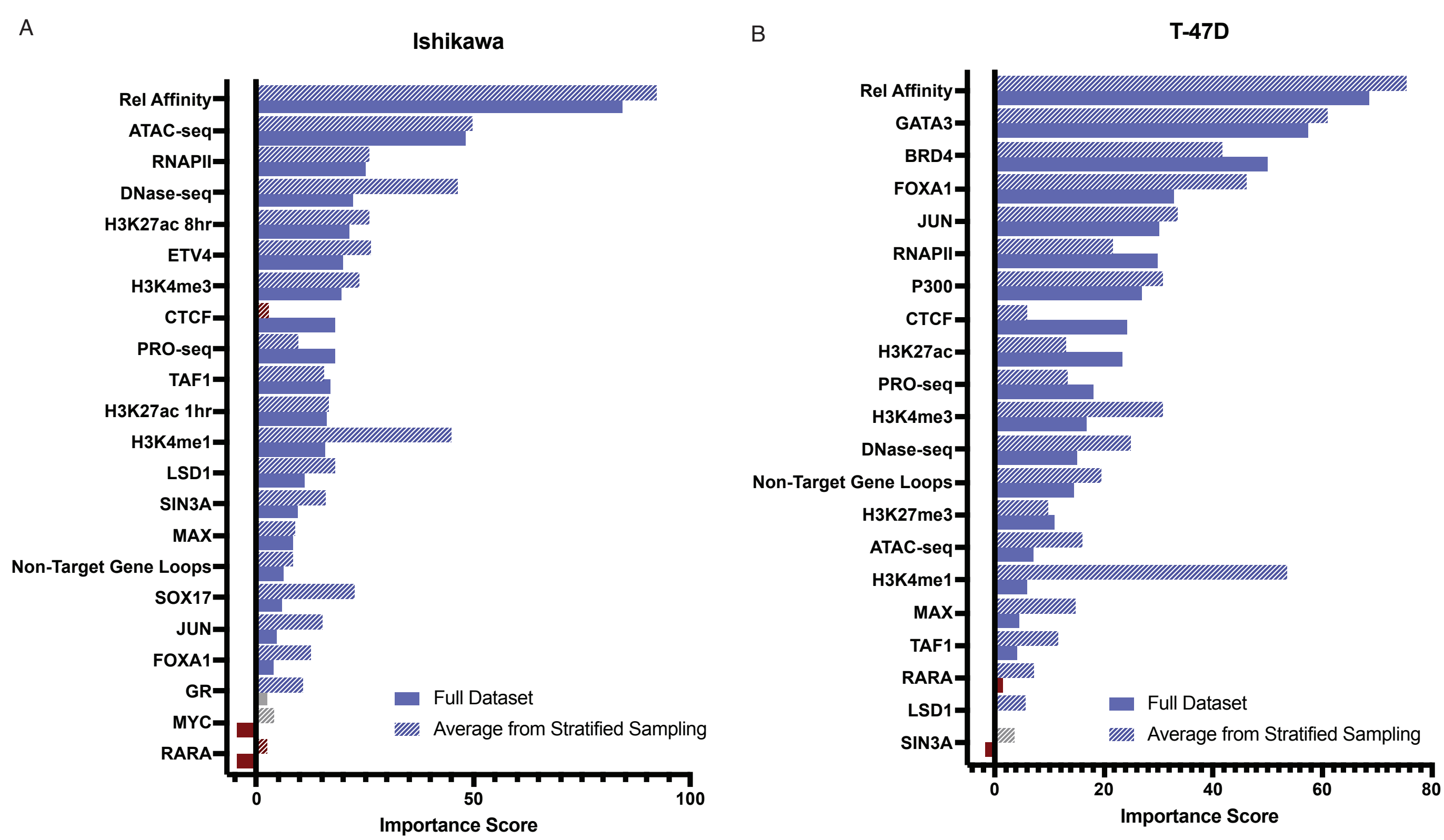

Supplemental Figure 1: Down sampling produces similar Boruta results as the full dataset for ERE binding prediction. Bar graph shows Boruta importance score from using full dataset (solid bars) compared to average importance score from down-sampled data (hashed bars) in A) Ishikawa and B) T-47D. The color of the bars indicates the decision; blue indicates confirmed in full data set Boruta run or confirmed in 80% or more of down-sampled Boruta runs, gray indicates tentative in full data set Boruta run or confirmed in between 50 and 80% of down-sampled Boruta runs, and red indicates rejected in full data set Boruta run or confirmed in less than 50% of down-sampled Boruta runs. This figure is related to Figure 2.

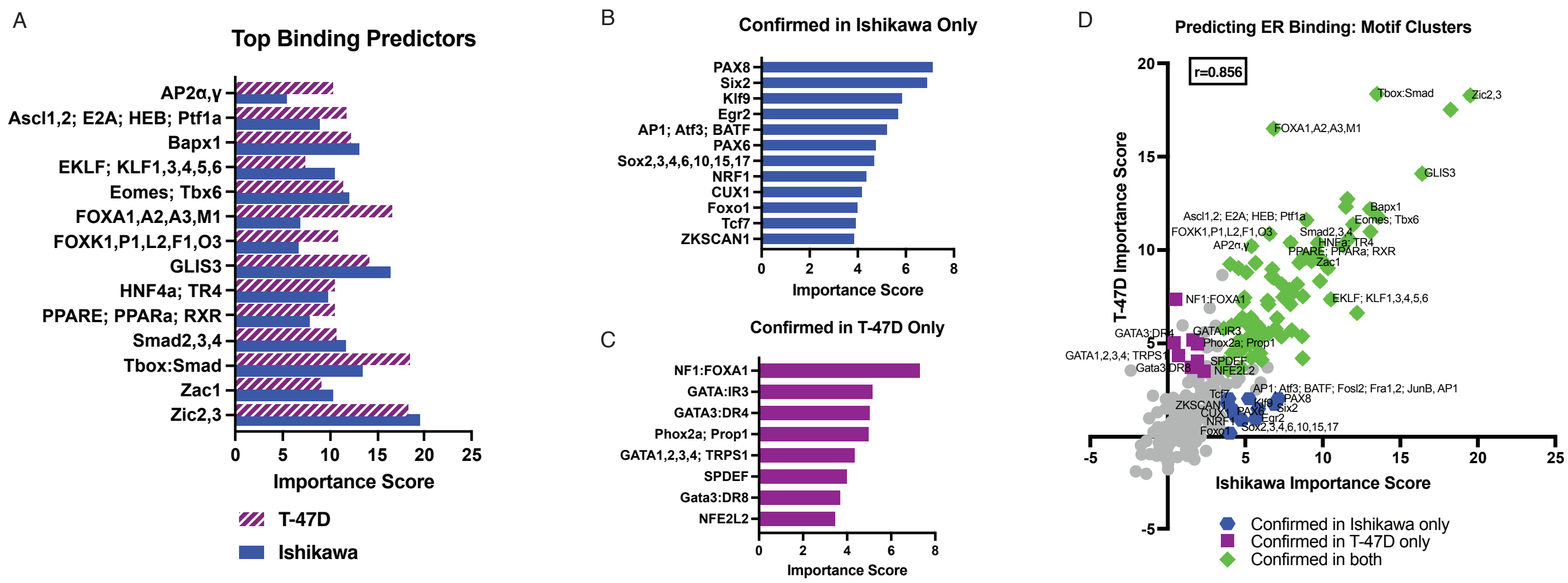

Supplemental Figure 2: Motif analysis reveals that top motif predictors are shared between cell lines. A) Bar chart shows the Boruta importance scores of the top scoring motif clusters for predicting bound EREs at an ERE in Ishikawa (blue) and T-47D (purple). Boruta importance scores are represented for motif clusters uniquely confirmed to be important in predicting bound EREs in B) Ishikawa and C) T-47D. D) Scatter plots show the comparison of Boruta importance scores between cell lines in predicting bound EREs from the motif cluster data set, with a Pearson correlation coefficient of 0.856. Green diamonds are clusters confirmed as important in predicting bound EREs by Boruta in both cell lines, blue hexagons are clusters confirmed only in Ishikawa, and purple squares are clusters confirmed only in T-47D. This figure is related to Figure 2.

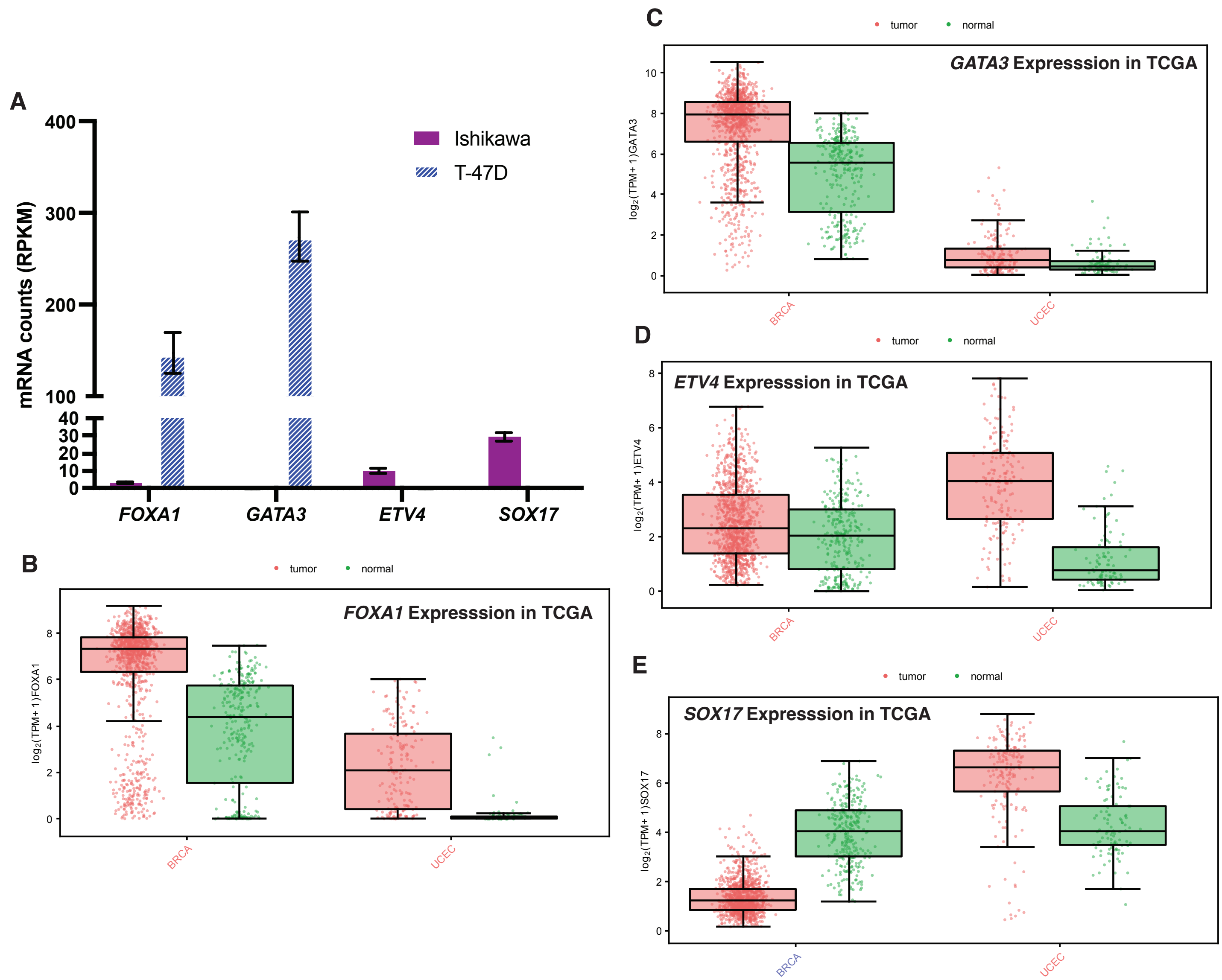

Supplemental Figure 3: Cell type-specific transcription factors are expressed more highly in their corresponding tissue type. A) Bar graph shows mRNA expression as reads per kilobase of transcript per million mapped reads for *FOXA1, GATA3, ETV4,* and *SOX17* in Ishikawa and T-47D cells. Boxplots show the expression of B) *FOXA1,* C) *GATA3*, D) *ETV4*, and E) *SOX17* in $\log_2$(TPM+1) in breast cancer samples (red, far left), breast normal tissue samples (green, second from left), endometrial cancer samples (red, second from right), and endometrial normal tissue samples (green, far right), created using GEPIA3 (Kang et al. 2025). This figure is related to Figure 2.

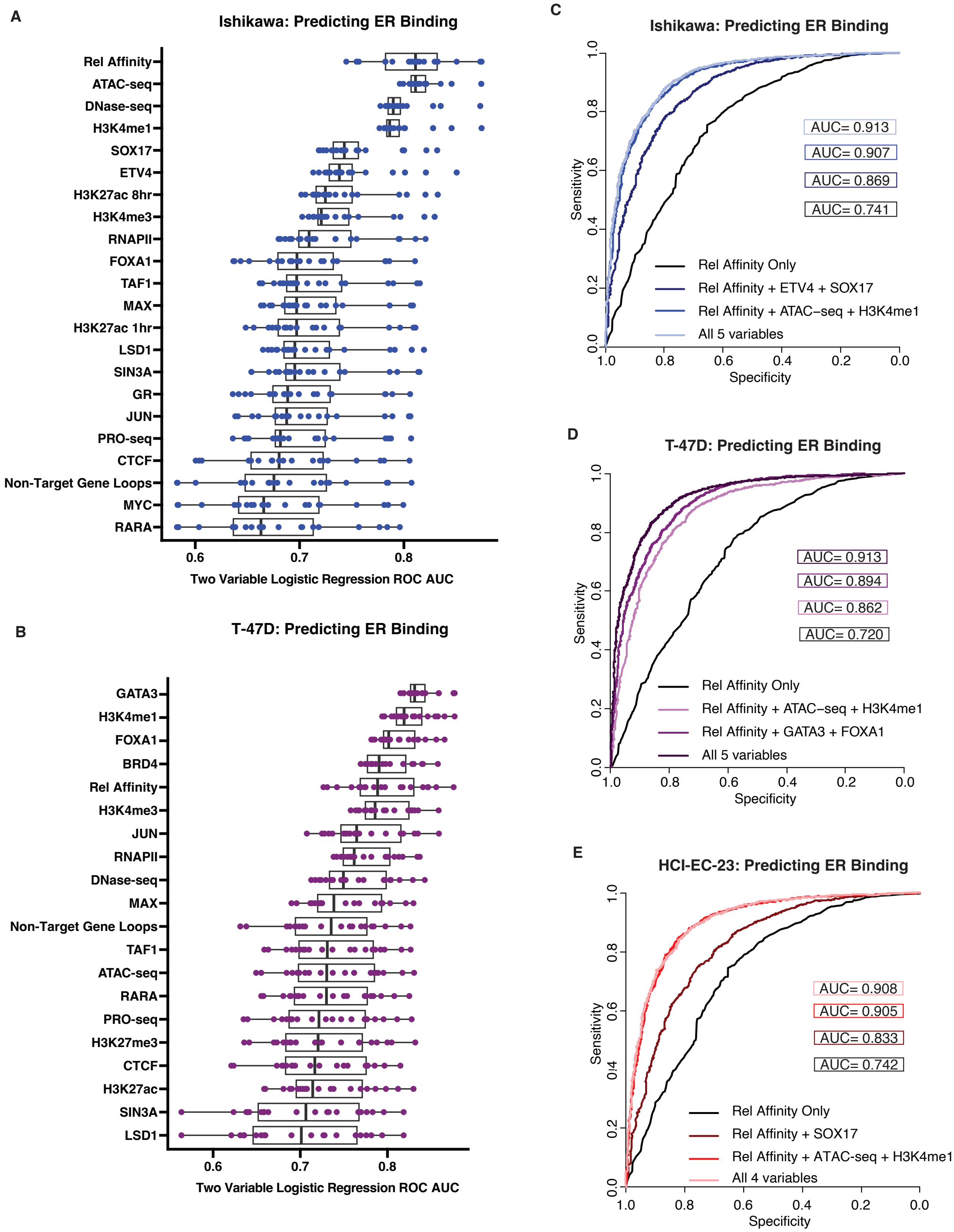

Supplemental Figure 4: Multivariable logistic regression models predict bound EREs with high sensitivity and high specificity. Ranked boxplots for each factor show the ROC AUC for all possible two variable logistic regression models predicting bound EREs in A) Ishikawa and B) T-47D that include that factor, i.e. the relative affinity boxplot is for all two variable logistic regression models containing relative affinity as one of the variables. Graphs show four different ROC curves, one using only relative affinity as a variable, one using relative affinity plus ATAC-seq and H3K4me1 ChIP-seq, one using relative affinity and cell line-specific transcription factor ChIP-seq datasets, and one using all variables from all other models for predicting bound EREs in C) Ishikawa, D) T-47D, and E) HCI-EC-23. AUC values are in boxes with colors corresponding to their ROCs.

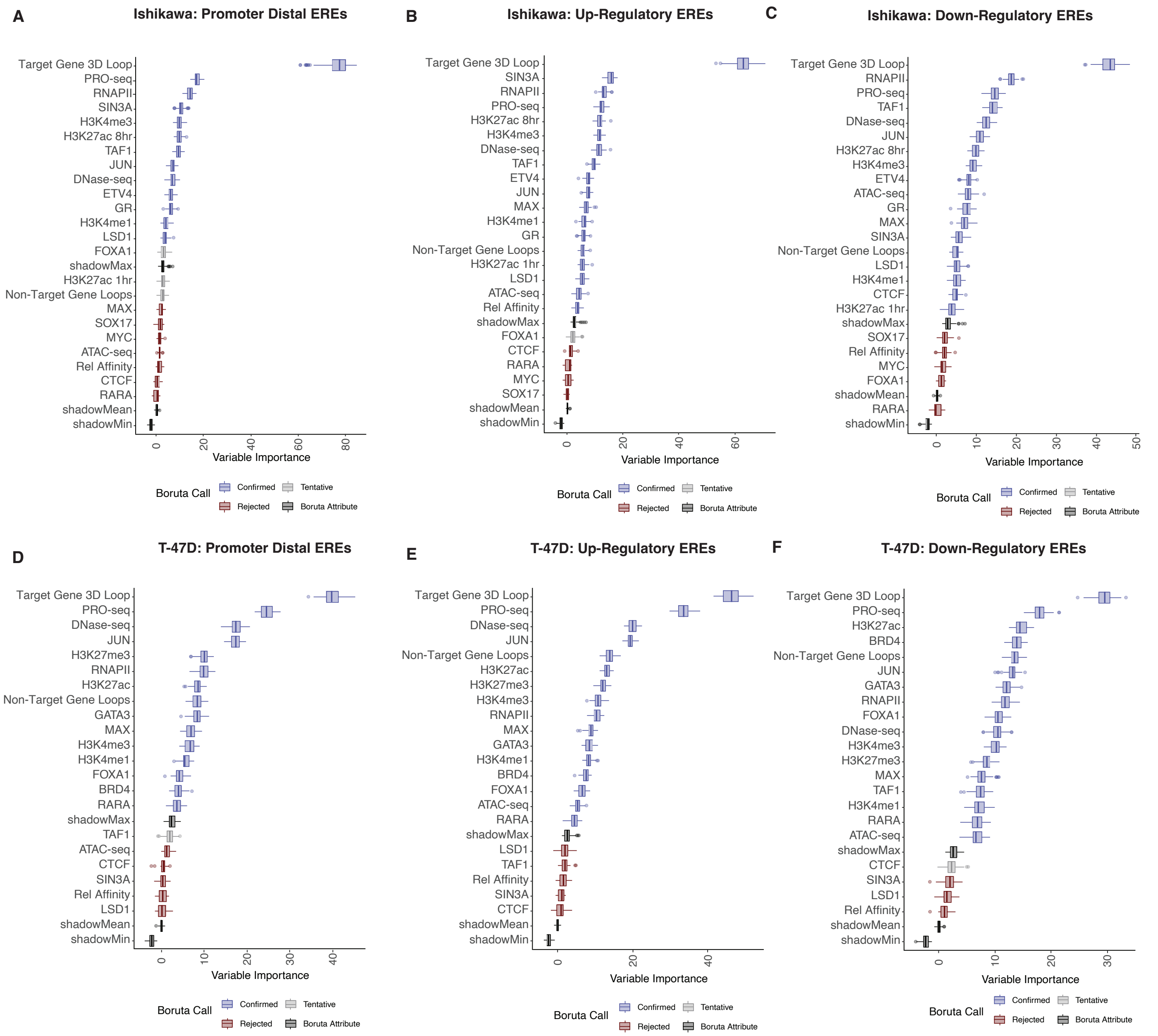

Supplemental Figure 5: Gene distance and gene regulation direction minimally impact regulatory ERE prediction. Boxplots show the ranked factor importance for predicting A) promoter-distal regulatory EREs versus non-regulatory EREs in Ishikawa, B) regulatory EREs associated with up-regulated genes versus non-regulatory EREs in Ishikawa, C) regulatory EREs associated with down-regulated genes versus non-regulatory EREs in Ishikawa, D) promoter-distal regulatory EREs versus non-regulatory EREs in T-47D, E) regulatory EREs associated with up-regulated genes versus non-regulatory EREs in T-47D, and F) regulatory EREs associated with down-regulated genes versus non-regulatory EREs in T-47D. Colors indicate Boruta determination of importance; blue is confirmed, light gray is tentative, red is rejected, and dark gray is a Boruta attribute. This figure is related to Figure 3.

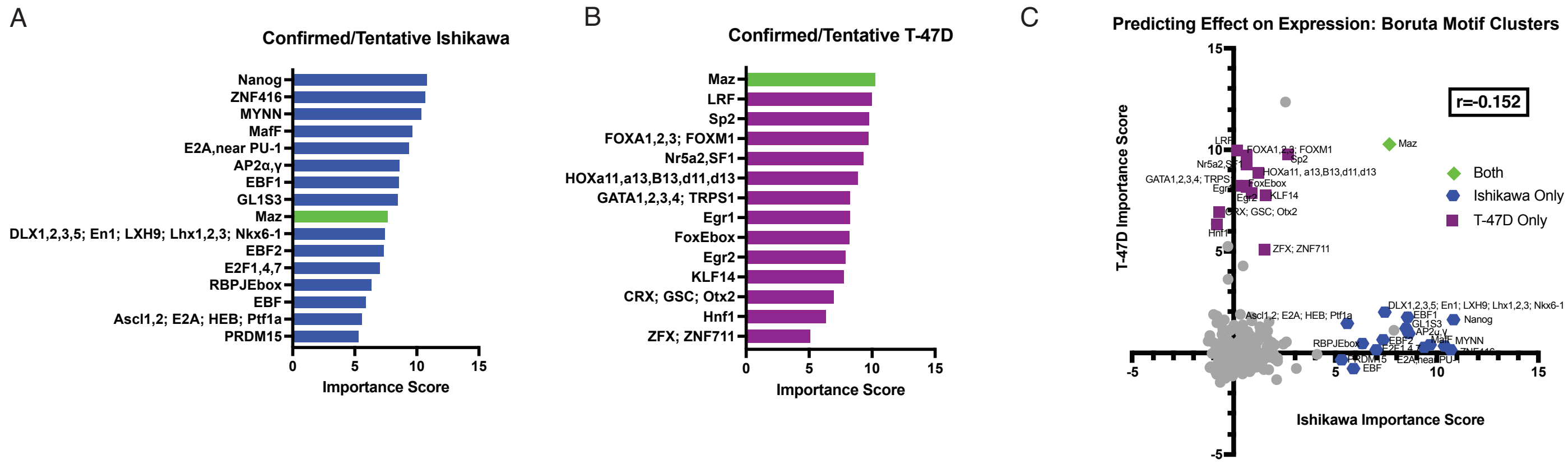

Supplemental Figure 6: Regulatory EREs are poorly predicted using motif analysis. Bar charts show motif clusters determined to be either confirmed or tentative by Boruta in being important for predicting regulatory EREs in A) Ishikawa or B) T-47D. Maz is the only motif confirmed important in both cell lines and is marked in green; all other motif clusters were uniquely determined to be confirmed or tentative in either Ishikawa (blue) or T-47D (purple). C) Scatter plots show the comparison of Boruta importance scores in predicting regulatory EREs from the motif cluster data set, with Pearson correlation coefficient of -0.152. The green diamond is the cluster confirmed as important in predicting regulatory EREs by Boruta in both cell lines, blue hexagons are clusters confirmed or designated tentative only in Ishikawa, and purple squares are clusters confirmed or designated tentative only in T-47D. This figure is related to Figure 3.

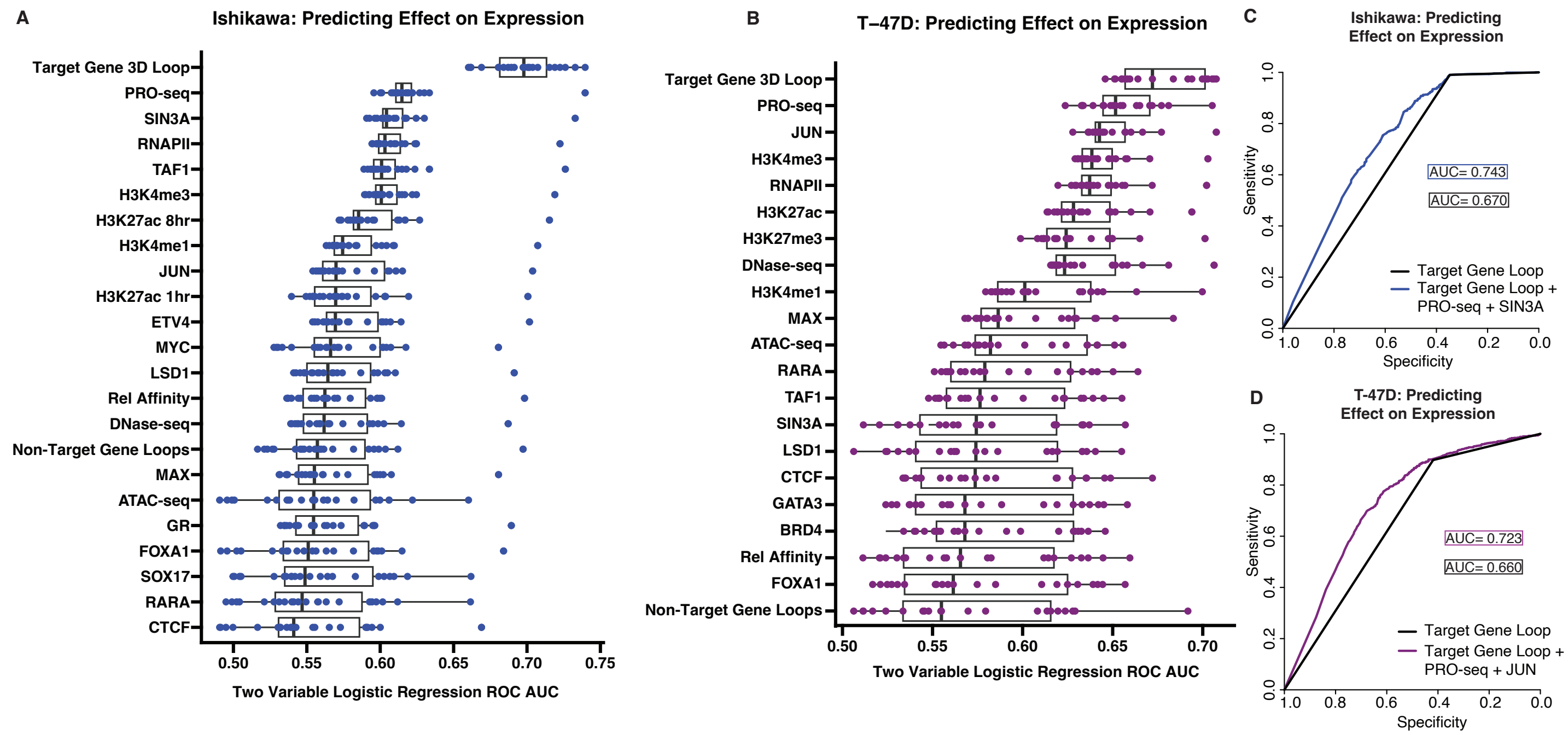

Supplemental Figure 7: Multivariable logistic regression models predict regulatory EREs with high specificity, but low sensitivity. Ranked boxplots for each factor show the ROC AUC for all possible two variable logistic regression models for predicting regulatory EREs that include that factor in A) Ishikawa and B) T-47D. Graphs show two different ROC curves, one using only Target Gene 3D Loop as a variable and one using Target Gene 3D Loop plus PRO-seq and a cell line-specific transcription factor ChIP-seq for predicting regulatory EREs in C) Ishikawa and D) T-47D. AUC values are in boxes with colors corresponding to their ROCs. This figure is related to Figure 3.

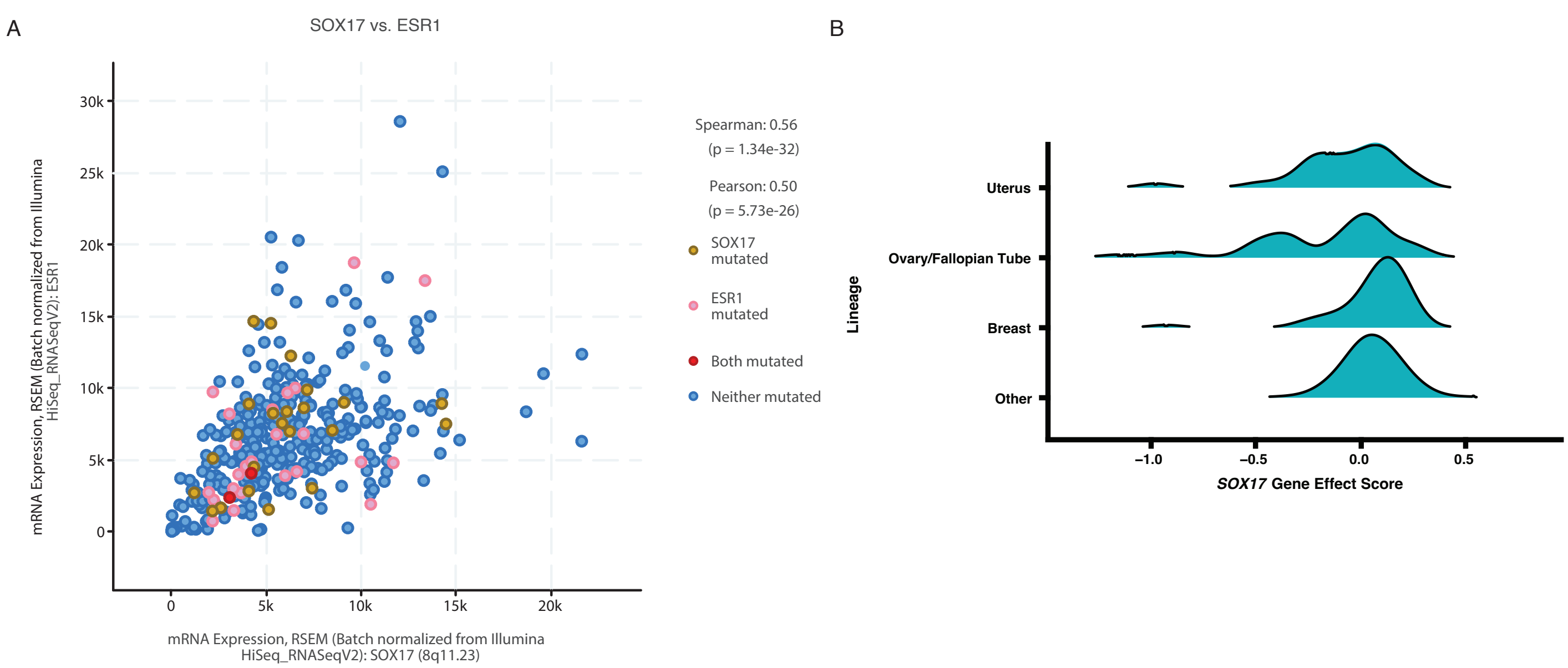

Supplemental Figure 8: SOX17 expression in patient data samples from The Cancer Genome Atlas. A)Scatter plot shows expression of *SOX17* versus the expression of *ESR1* in endometrial tumors using mRNA expression z-scores relative to diploid samples, created using cBioPortal (Cerami et al. 2012; Gao et al. 2013; de Bruijn et al. 2023). B) Density plot shows the gene effect score as calculated by DepMap (Arafeh et al. 2025) of SOX17 in cancer models from uterus, ovary/fallopian tube, breast, and all other lineages.

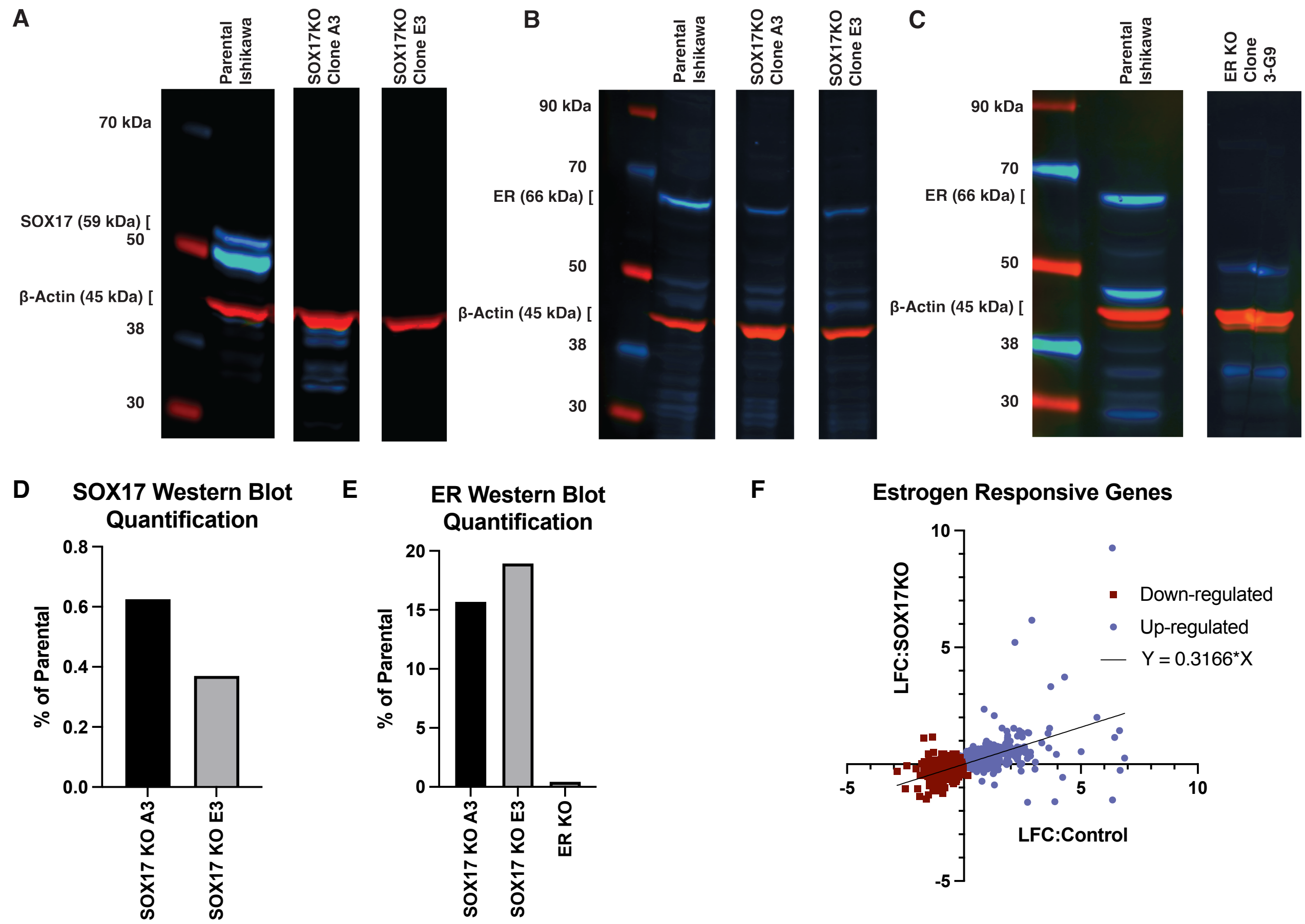

Supplemental Figure 9: SOX17 and ER KO affect protein levels. A) Western blot shows absence of SOX17 protein in SOX17 KO lines (SOX17 in blue, beta actin in red). B) Western blot shows reduced ER protein in SOX17 KO lines (ER in blue, beta actin in red). C) Western blot shows absence of ER protein in ER KO line (ER in blue, beta actin in red). Bar graphs show Western blot quantification for D) panel A and E) panels B and C. F) Scatter plot shows $\log_2$(fold change) of estrogen responsive genes in SOX17 KO lines compared to control lines, with linear regression line shown in black. This figure is related to Figure 6.

Supplemental Table 1: Datasets used in the Boruta analyses

Cell Line	Factor	Reference	GEO Accession
Ishikawa	ATAC-seq	(Vahrenkamp et al. 2018)	GSE109893
Ishikawa	CTCF	ENCODE (ENCODE Project Consortium 2012)	GSE32465
Ishikawa	DNase-seq	ENCODE (ENCODE Project Consortium 2012)	GSE32970
Ishikawa	ETV4	ENCODE (ENCODE Project Consortium 2012)	GSE32465
Ishikawa	FOXA1	ENCODE (ENCODE Project Consortium 2012)	GSE32465
Ishikawa	GR	(Vahrenkamp et al. 2018)	GSE109893
Ishikawa	H3K27ac 1hr	(Vahrenkamp et al. 2018)	GSE109893
Ishikawa	H3K27ac 8hr	(Vahrenkamp et al. 2018)	GSE109893
Ishikawa	H3K4me1	(Ginley-Hidinger et al. 2024)	GSE227241
Ishikawa	H3K4me3	(Ginley-Hidinger et al. 2024)	GSE227241
Ishikawa	Hi-ChIP	(Ginley-Hidinger et al. 2024)	GSE227242
Ishikawa	JUN	(Ginley-Hidinger et al. 2024)	GSE227241
Ishikawa	LSD1	(Ginley-Hidinger et al. 2024)	GSE227241
Ishikawa	MAX	(Ginley-Hidinger et al. 2024)	GSE227241
Ishikawa	MYC	(Ginley-Hidinger et al. 2024)	GSE227241
Ishikawa	PRO-seq	(Ginley-Hidinger et al. 2024)	GSE227243
Ishikawa	RARA	(Ginley-Hidinger et al. 2024)	GSE227241
Ishikawa	Rel Affinity	This work	
Ishikawa	RNAPII	ENCODE (ENCODE Project Consortium 2012)	GSE32465
Ishikawa	SIN3A	(Ginley-Hidinger et al. 2024)	GSE227241
Ishikawa	SOX17	This work	GSE 312323
Ishikawa	TAF1	(Ginley-Hidinger et al. 2024)	GSE227241
T-47D	ATAC-seq	(Arnesen et al. 2021)	GSE148278
T-47D	BRD4	(Shu et al. 2016)	GSE63581
T-47D	CTCF	(Gertz et al. 2013)	GSE32465
T-47D	DNase-seq	ENCODE (ENCODE Project Consortium 2012)	GSE32970
T-47D	FOXA1	ENCODE (ENCODE Project Consortium 2012)	GSE32465
T-47D	GATA3	ENCODE (ENCODE Project Consortium 2012)	GSE32465
T-47D	H3K27ac	(Carleton et al. 2020)	GSE147141
T-47D	H3K27me3	(Ginley-Hidinger et al. 2024)	GSE227241
T-47D	H3K4me1	(Ginley-Hidinger et al. 2024)	GSE227241
T-47D	H3K4me3	(Ginley-Hidinger et al. 2024)	GSE227241
T-47D	Hi-ChIP	(Ginley-Hidinger et al. 2024)	GSE227242
T-47D	JUN	(Ginley-Hidinger et al. 2024)	GSE227241
T-47D	LSD1	(Ginley-Hidinger et al. 2024)	GSE227241
T-47D	MAX	(Ginley-Hidinger et al. 2024)	GSE227241
T-47D	PRO-seq	(Ginley-Hidinger et al. 2024)	GSE227243
T-47D	RARA	(Ginley-Hidinger et al. 2024)	GSE227241
T-47D	ERE Rel Affinity	This work	
T-47D	RNAPII	(Ginley-Hidinger et al. 2024)	GSE227241

T-47D	SIN3A	(Ginley-Hidinger et al. 2024)	GSE227241
T-47D	TAF1	(Ginley-Hidinger et al. 2024)	GSE227241

Supplemental Table 6: Primer Sequences

Primer Name	Sequence
3XERE PCR Forward Primer	TAAGCAAAGCTTCGAGCTCTTACGCGTGCTA
3XERE PCR Reverse Primerr	TAAGCAATCGATCGAGCTCTTACGCGTGCTA
CRISPR Screen Genomic DNA PCR Amplification Forward Primer	TCGTCGGCAGCGTCAGATGTGTATAAGAGA CAGTTGTGGAAAGGACGAAACACCG
CRISPR Screen Genomic DNA PCR Amplification Reverse Primer	GTCTCGTGGGCTCGGAGATGTGTATAAGAG ACAGCCAATTCCCACTCCTTTCAAGACCT
Index 1 Read Adapter for CRISPR Screen	CAAGCAGAAGACGGCATACGAGAT[i7]GTC TCGTGGGCTCGG
Index 2 Read Adapter for CRISPR Screen	AATGATACGGCGACCACCGAGATCTACAC [i5]TCGTCGGCAGCGTC
SOX17 KO gRNA 1 Forward	CACCGGCCGAGTTGAGCAAGATGCT
SOX17 KO gRNA 1 Reverse	AAACAGCATCTTGCTCAACTCGGCC
SOX17 KO gRNA 2 Forward	CACCGAGGGCGAGTCCCGTATCCGG
SOX17 KO gRNA 2 Reverse	AAACCCGGATACGGGACTCGCCCTC
ER KO gRNA Forward	CACCGGGGATACGAAAAGACCGAAG
ER KO gRNA Reverse	AAACCTTCGGTCTTTTCGTATCCCC

Supplemental References

Arafeh R, Shibue T, Dempster JM, Hahn WC, Vazquez F. 2025. The present and future of the Cancer Dependency Map. *Nat Rev Cancer* **25**: 59–73.

Arnesen S, Blanchard Z, Williams MM, Berrett KC, Li Z, Oesterreich S, Richer JK, Gertz J. 2021. Estrogen receptor alpha mutations in breast cancer cells cause gene expression changes through constant activity and secondary effects. *Cancer Res* **81**: 539–551.

Carleton JB, Ginley-Hidinger M, Berrett KC, Layer RM, Quinlan AR, Gertz J. 2020. Regulatory sharing between estrogen receptor alpha bound enhancers. *Nucleic Acids Res* **48**: 6597–6610.

Cerami E, Gao J, Dogrusoz U, Gross BE, Sumer SO, Aksoy BA, Jacobsen A, Byrne CJ, Heuer ML, Larsson E et al. 2012. The cBio cancer genomics portal: an open platform for exploring multidimensional cancer genomics data. *Cancer Discov* **2**: 401–404.

de Bruijn I, Kundra R, Mastrogiacomo B, Tran TN, Sikina L, Mazor T, Li X, Ochoa A, Zhao G, Lai B et al. 2023. Analysis and Visualization of Longitudinal Genomic and Clinical Data from the AACR Project GENIE Biopharma Collaborative in cBioPortal. *Cancer Res* **83**: 3861–3867.

ENCODE Project Consortium. 2012. An integrated encyclopedia of DNA elements in the human genome. *Nature* **489**: 57–74.

Gao J, Aksoy BA, Dogrusoz U, Dresdner G, Gross B, Sumer SO, Sun Y, Jacobsen A, Sinha R, Larsson E et al. 2013. Integrative analysis of complex cancer genomics and clinical profiles using the cBioPortal. *Sci Signal* **6**: pl1.

Gertz J, Savic D, Varley KE, Partridge EC, Safi A, Jain P, Cooper GM, Reddy TE, Crawford GE, Myers RM. 2013. Distinct properties of cell-type-specific and shared transcription factor binding sites. *Mol Cell* **52**: 25–36.

Ginley-Hidinger M, Abewe H, Osborne K, Richey A, Kitchen N, Mortenson KL, Wissink EM, Lis J, Zhang X, Gertz J. 2024. Cis-regulatory control of transcriptional timing and noise in response to estrogen. *Cell Genom* **4**: 100542.

Guhathakurta D. 2006. Computational identification of transcriptional regulatory elements in DNA sequence. *Nucleic Acids Res* **34**: 3585–3598.

Hall JM, McDonnell DP. 1999. The estrogen receptor beta-isoform (ERbeta) of the human estrogen receptor modulates ERalpha transcriptional activity and is a key regulator of the cellular response to estrogens and antiestrogens. *Endocrinology* **140**: 5566–5578.

Heinz S, Benner C, Spann N, Bertolino E, Lin YC, Laslo P, Cheng JX, Murre C, Singh H, Glass CK. 2010. Simple Combinations of Lineage-Determining Transcription Factors Prime cis-Regulatory Elements Required for Macrophage and B Cell Identities. *Mol Cell* **38**: 576–589.

Hertz GZ, Stormo GD. 1999. Identifying DNA and protein patterns with statistically significant alignments of multiple sequences. *Bioinformatics* **15**: 563–577.

Kang Y-J, Pan L, Liu Y, Rong Z, Liu J, Liu F. 2025. GEPIA3: Enhanced drug sensitivity and interaction network analysis for cancer research. *Nucleic Acids Res* **53**: W283–W290.

Kuhn M, Wing J, Weston S, Williams A, Keefer C, Engelhardt A, Cooper T, Mayer Z, Kenkel B, Team RC. 2020. Package ‘caret’. *The R Journal* **223**: 48.

Kursa MB, Rudnicki WR. 2010. Feature Selection with the Boruta Package. *J Stat Softw* **36**: 1 – 13.

Li W, Xu H, Xiao T, Cong L, Love MI, Zhang F, Irizarry RA, Liu JS, Brown M, Liu XS. 2014. MAGeCK enables robust identification of essential genes from genome-scale CRISPR/Cas9 knockout screens. *Genome Biol* **15**: 554.

Quinlan AR, Hall IM. 2010. BEDTools: a flexible suite of utilities for comparing genomic features. *Bioinformatics* **26**: 841–842.

R Core Team. 2016. R: A language and environment for statistical computing. R Foundation for Statistical Computing, Vienna, Austria. *http://www R-project org/*.

Robin X, Turck N, Hainard A, Tiberti N, Lisacek F, Sanchez J-C, Müller M, Siegert S, Doering M, Billings Z. 2011. pROC: an open-source package for R and S+ to analyze and compare ROC curves. *BMC Bioinformatics* **12**: 77.
Rodriguez AC, Vahrenkamp JM, Berrett KC, Clark KA, Guillen KP, Scherer SD, Yang CH, Welm BE, Janat-Amsbury MM, Graves BJ et al. 2020. ETV4 Is Necessary for Estrogen Signaling and Growth in Endometrial Cancer Cells. *Cancer Res* **80**: 1234–1245.
Roesch A, Fukunaga-Kalabis M, Schmidt EC, Zabierowski SE, Brafford PA, Vultur A, Basu D, Gimotty P, Vogt T, Herlyn M. 2010. A Temporarily Distinct Subpopulation of Slow-Cycling Melanoma Cells Is Required for Continuous Tumor Growth. *Cell* **141**: 583–594.
Rush CM, Blanchard Z, Polaski JT, Osborne KS, Osby K, Vahrenkamp JM, Yang C-H, Lum DH, Hagan CR, Leslie KK et al. 2022. Characterization of HCI-EC-23 a novel estrogen- and progesterone-responsive endometrial cancer cell line. *Sci Rep* **12**: 19731.
Sanjana NE, Shalem O, Zhang F. 2014. Improved vectors and genome-wide libraries for CRISPR screening. *Nat Methods* **11**: 783–784.
Shu S, Lin CY, He HH, Witwicki RM, Tabassum DP, Roberts JM, Janiszewska M, Jin Huh S, Liang Y, Ryan J. 2016. Response and resistance to BET bromodomain inhibitors in triple-negative breast cancer. *Nature* **529**: 413–417.
Stewart SA, Dykxhoorn DM, Palliser D, Mizuno H, Yu EY, An DS, Sabatini DM, Chen IS, Hahn WC, Sharp PA et al. 2003. Lentivirus-delivered stable gene silencing by RNAi in primary cells. *RNA* **9**: 493–501.
Vahrenkamp JM, Yang C-H, Rodriguez AC, Almomen A, Berrett KC, Trujillo AN, Guillen KP, Welm BE, Jarboe EA, Janat-Amsbury MM et al. 2018. Clinical and Genomic Crosstalk between Glucocorticoid Receptor and Estrogen Receptor α In Endometrial Cancer. *Cell Rep* **22**: 2995–3005.
Williams RT, Guarecuco R, Gates LA, Barrows D, Passarelli MC, Carey B, Baudrier L, Jeewajee S, La K, Prizer B et al. 2020. ZBTB1 Regulates Asparagine Synthesis and Leukemia Cell Response to L-Asparaginase. *Cell Metab* **31**: 852–861.e856.
